# Staff perception of Lean, care-giving, thriving and exhaustion: a longitudinal study in primary care

**DOI:** 10.1186/s12913-019-4502-6

**Published:** 2019-09-09

**Authors:** Monica Kaltenbrunner, Lars Bengtsson, Svend Erik Mathiassen, Hans Högberg, Maria Engström

**Affiliations:** 1 Department of Occupational Health Sciences and Psychology, Faculty of Health and Occupational Studies, University of Gävle, 801 76 Gävle, Sweden; 2 Department of Industrial Management, Industrial Design and Mechanical Engineering, Faculty of Engineering and Sustainable Development, University of Gävle, 801 76 Gävle, Sweden; 3 Department of Caring Science, Faculty of Health and Occupational Studies, University of Gävle, 801 76 Gävle, Sweden; 4 Department of Public Health and Caring Sciences, Uppsala University, Uppsala, Sweden; 5 Nursing Department, Medicine and Health College, Lishui University, Lishui, China

**Keywords:** COPSOQ, JD-R theory, Linear mixed model, LiHcQ Lean in healthcare questionnaire, Quality of care, Thriving, Exhaustion

## Abstract

**Background:**

Lean is commonly adopted in healthcare to increase quality of care and efficiency. Few studies of Lean involve staff-related outcomes, and few have a longitudinal design. Thus, the aim was to examine the extent to which changes over time in Lean maturity are associated with changes over time in care-giving, thriving and exhaustion, as perceived by staff, with a particular emphasis on the extent to which job demands and job resources, as perceived by staff, have a moderated mediation effect.

**Method:**

A longitudinal study with a correlational design was used. In total, 260 staff at 46 primary care units responded to a web survey in 2015 and 2016. All variables in the study were measured using staff ratings. Ratings of Lean maturity reflect participants’ judgements regarding the entire unit; ratings of care-giving, thriving, exhaustion and job demands and resources reflect participants’ judgements regarding their own situation.

**Results:**

First, over time, increased Lean maturity was associated with increased staff satisfaction with their care-giving and increased thriving, mediated by increased job resources. Second, over time, increased Lean maturity was associated with decreased staff exhaustion, mediated by decreased job demands. No evidence was found showing that job demands and job resources had a moderated mediation effect.

**Conclusion:**

The results indicate that primary care staff may benefit from working in organizations characterized by high levels of Lean maturity and that caregiving may also be improved as perceived by staff.

**Electronic supplementary material:**

The online version of this article (10.1186/s12913-019-4502-6) contains supplementary material, which is available to authorized users.

## Background

Use of Lean in healthcare has spread [1, 2], the aim being to increase quality of care [3] and efficiency [4]. The actual outcomes of Lean adoption seems, however, less clear [5]. Concerning Lean and staff health and working conditions, in two review papers mainly including studies from auto and manufacturing industries [6, 7] and in a study from the manufacturing industry [8], the findings varied, although most suggested Lean to have negative effects. In healthcare, reviews have reported improved staff and patient satisfaction as well as decreased errors and patient mortality after adoption of Lean [1, 9], thus, the possibility for positive publication bias [10] shall be considered. On the other hand, one study, found that Lean can contribute to increased stress and exhaustion among healthcare staff [11]. Previous research suggests that further attention needs to be given to outcomes such as quality of care [1, 12], staff engagement [11], staff and patient satisfaction [1], as well as health and working conditions [1, 4, 5, 9]. Moreover, there is a lack of longitudinal studies and thorough assessments of Lean [1].

No shared definition of Lean exists [1, 13], but several descriptions have been offered [14–17]. The present study is based on Liker’s [14] description, according to which Lean involves 14 principles combined in a 4P model: Philosophy, Processes, People and partners, and Problem-solving. Philosophy refers to, for instance, having long-term goals, focusing on customer needs and having engaged staff. Processes includes for instance creating flow in the processes which can be achieved by value stream mapping, waste reduction and standardization. Waste can be activities that do not add value for customers, such as errors or waiting time. People and partners refers to, for example, working in multidisciplinary teams and showing respect for people. Problem-solving includes staff continuously improving care processes. Because staff are involved in problem-solving and decision-making, they have opportunities to be engaged, learn, grow and develop [14]. According to Liker [14], if set goals are to be achieved, Lean needs to be adopted system wide, involve all Lean principles and all staff members. In the present study, the extent to which this is achieved is termed Lean maturity [18]. Review papers in healthcare have shown that Lean has most often been only partially adopted [1, 2, 9].

### Lean and working conditions

In the present study, we hypothesized that there would be a relationship between Lean maturity, as perceived by staff, and different outcomes, also perceived by staff, and we asked whether working conditions might have a moderated mediation effect. According to the Job Demands-Resources (JD-R) theory proposed by Bakker and Demerouti [19], working conditions involve all types of job characteristics. In the theory, working conditions are divided into job demands or job resources. Job demands can be high workload and work pace, working overtime, role overload, poor environmental conditions and emotional demands [19]. Job resources can be having multiple skills, autonomy, performance feedback, possibilities for development [19], support, role clarity, influence and recognition [20]. The theory suggests dual processes, where job demands are linked to exhaustion and job resources to engagement, motivation and job performance [19]. A literature overview including for instance studies in restaurants and healthcare, showed that job resources contributed to engagement and consequently to improved job performance [21], whereas a study involving staff from human services, industry and transport reported that having few resources seemed to be related to staff disengagement [22]. Leiter and Bakker suggested that having job resources could also enhance thriving at work [23]. Empirical studies from the healthcare sector have shown that having high job demands contributed to increased exhaustion [24, 25]; however, in a study of knowledge workers (e.g., professors, scientists, engineers, teachers, managers, secretaries and physicians), high job demands improved job performance [26]. Apart from the dual processes, in the JD-R theory, Bakker and Demerouti [19] also suggested that an interaction effect exists between job demands and resources, claiming that “job resources can buffer the impact of job demands on strain” ([19], p., 274) and that “job resources particularly influence motivation when job demands are high” ([19], p., 275). Support for the suggested interaction effect has been offered; for instance, a study of an electronics company [27] and a study in eldercare services [28] revealed that job resources could buffer the effect of job demands. As described above, job resources have been related to job performance and engagement; job demands have also been shown to contribute to disengagement [22], and according to a meta-analysis by Gilboa et al., to decreased job performance [29]. An interaction effect was also described in a study by Bakker et al. [30] of occupational health service providers, where job resources were associated with task enjoyment and commitment, especially when job demands were high.

**Fig. 1:**
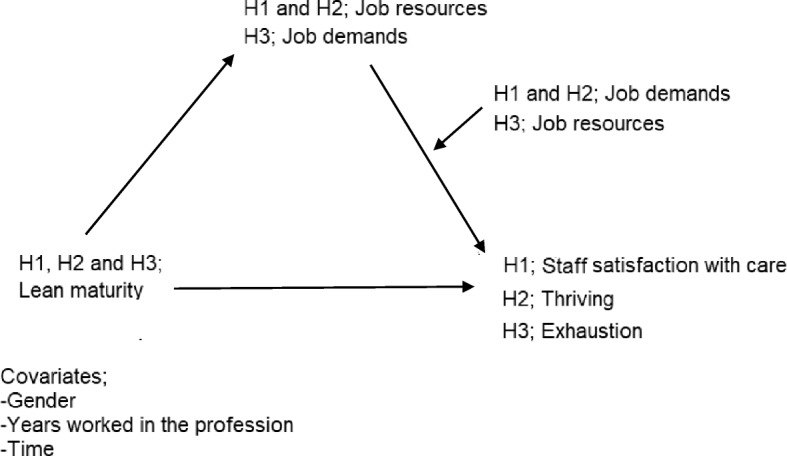
Model of the hypothesized pathways between Lean maturity, working conditions as moderating mediators, and the three outcomes, which in H1 were staff satisfaction with care, in H2, thriving and in H3, exhaustion

Working conditions have also been considered in studies of Lean. For instance, a study of predominantly primary care showed that Lean adoption resulted in work improvement and increased staff responsibility, and that introducing multiprofessional teamwork resulted in a decrease in reported hierarchy on the units [31]. Dellve et al.’s [32] longitudinal study, included hospitals, revealed that when Lean was comprehensively adopted, staff perceived that resources increased and that demands decreased [32]. However, Leijen-Zeelenberg et al. [33] found a small, but significant deterioration of staff autonomy and participation when Lean was adopted at an outpatient clinic. From this perspective, Lean exists on an organizational level but influence staff on the individual level. Thus, the present study will include these different levels, an approach that Bakker and Demerouti [19] called for. We hypothesize three pathways along which Lean maturity will influence staff perception of their own care-giving, thriving and exhaustion, taking into account was that working conditions would likely show an interaction effect [19] in these associations. Figure 1 illustrates the hypothesized pathways. In the next sections, the outcomes will be explicitly defined. We will also explain the hypothetical relationships between Lean and the three outcomes, and the reasons for believing that working conditions influence these relationships.

### Lean and staff satisfaction with care

We suggest that staff satisfaction with care, also termed care-giving, is indicative of quality of care. Earlier studies have demonstrated a relationship between Lean and quality of care. For instance, two longitudinal studies revealed that when Lean was adopted at a radiotherapy institute, errors decreased and the safety culture improved [12], and when adopted at a hospital, mortality and waiting times to operations decreased [34]. A case study in a hospital setting reported increased patient satisfaction after Lean adoption [35]. In contrast, two longitudinal studies, one at an outpatient clinic and one in primary care, showed no significant improvements in patient satisfaction [33, 36]. Further, working conditions have been shown to influence quality of care. For instance, several studies from the healthcare sector have shown that resources such as social support [37–39], involvement in decision-making [37] and having job control [38] were positively related to staff-rated quality of care. Demands referred to as workload have been negatively related to staff self-rated quality of care [37, 38]. Thereby, our first hypothesis (Fig. 1) was that there would be a relationship between staff-perceived Lean maturity and staff satisfaction with care, mediated by job resources, and that the link would be moderated by job demands.

### Lean and thriving

Thriving is a psychological state that can be sensed when individuals experience vitality and learning at work [40]. Sensing that one is thriving can help enhance career development and job performance [41]. To our knowledge, no studies to date have involved both Lean and thriving. However, it has been shown that thriving and engagement are related constructs with commonalities, and that they are mutually reinforcing [23]. It is likely that a person who has a sense of thriving will also feel engaged [23]. Staff engagement includes, according to Macey and Schneider [42], commitment, involvement, energy, enthusiasm, empowerment, taking initiative, willingness to accept changes and assuming responsibility. In a longitudinal study in primary care, engagement increased after Lean adoption [11], a finding also described in other studies focused on the healthcare sector [24, 31, 43]. Further, there might be a relationship between working conditions and thriving, as described by Leiter and Bakker [23]. In their study of occupational health services providers, Bakker et al. [30] demonstrated that working conditions in the form of high job demands combined with appropriate resources can contribute to thriving. Hence, our second hypothesis (Fig. 1) was that there would be a relationship between staff-perceived Lean maturity and thriving, mediated by job resources, and that the link would be moderated by job demands.

### Lean and exhaustion

Exhaustion, which is the core component of psychological stress-related burnout syndrome, involves having increased feelings of emotional exhaustion [44]. A relationship between Lean and exhaustion was described in a longitudinal study by Lindskog et al. [24]. They showed that, after Lean adoption, exhaustion increased significantly at one hospital, but not at the other participating hospital and municipality [24]. Another longitudinal study, in primary care, found that job stress and exhaustion increased after Lean adoption [11]. Further, a systematic review reported that experiencing demands, defined as having low control, high demands and high workload, may increase the risk of developing exhaustion [45]. In two additional studies, one including both hospitals and a municipality and one focused on teachers, high demands were positively related to exhaustion [24, 46]. However, a study of occupational health service providers showed that high demands may not always contribute to adverse outcomes, provided that resources are available [30]. In a study in healthcare, having resources – defined as having opportunities for development – was negatively related to exhaustion [24]. Thus, our third hypothesis (Fig. 1) was that there would be a relationship between staff-perceived Lean maturity and exhaustion, mediated by job demands, and that the link would be moderated by job resources.

Thus, the aim was to examine the extent to which changes over time in Lean maturity are associated with changes over time in care-giving, thriving and exhaustion, as perceived by staff, with a particular emphasis on the extent to which job demands and job resources, as perceived by staff, have a moderated mediation effect.

*Hypothesis H1:* Assessing changes over time, increased Lean maturity is associated with increased staff perception of satisfaction with their care-giving, and the association is mediated by increased job resources. The link between job resources and staff perception of care-giving is, in turn, moderated by job demands.

*Hypothesis H2:* Assessing changes over time, increased Lean maturity is associated with increased staff perception of thriving, and the association is mediated by increased job resources. The link between job resources and staff perception of thriving is, in turn, moderated by job demands.

*Hypothesis H3:* Assessing changes over time, increased Lean maturity is associated with decreased staff perception of exhaustion, and the association is mediated by decreased job demands. The link between job demands and staff perception of exhaustion is, in turn, moderated by job resources.

## Methods

### Design, setting and study sample

The study had a longitudinal and correlational design. A convenience sample included all primary care units in a region in central Sweden. In Sweden, the primary care system is responsible for providing basic healthcare, including prevention, advice and treatment [47]. In total 52 public and privately owned healthcare units were asked to participate, 42 accepted. Only four of these units were privately owned, and therefore one of the largest private healthcare providers in Sweden was approached; 6 of its 85 units agreed to participate. Thus, a total of 48 units agreed to participate. For units to be eligible, they must have adopted Lean to some extent. No information was collected concerning when or how the units adopted Lean. The study is part of a larger research project focusing on Lean in primary care; cross-sectional data have been used in another study [18], and future studies will be conducted.

The inclusion criterion for staff was having worked at the present unit at least 3 months prior to data collection. All staff working at the included units received written study information and an Internet-based survey. Two reminders and later a paper version of the questionnaire were sent to non-responders. At data collection time (T) 1, spring 2015, 1040 staff were eligible from 48 units; 481 questionnaires were returned (response rate 46%). Of the 481 participants at T1, 406 were eligible at T2 1 year later (Table 1). The number responding at both T1 and T2 decreased to *n* = 260, from 46 units.

**Table 1:**
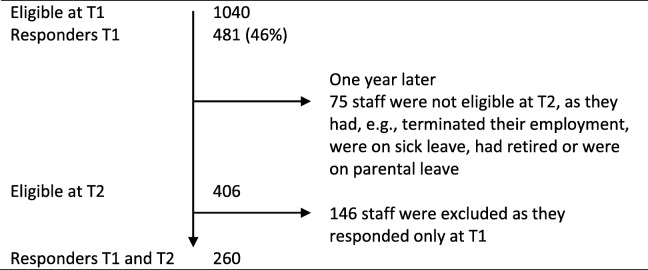
Flowchart of eligible staff members and participants

T1 First data collection (2015), T2 Second data collection (2016)

Most participants were women (87%) with a mean age of 50 years (SD10.3) and worked in public non-profit units (85%) (Table 2). The educational level of 3–5 years at university was most common. When comparing those excluded from the present study because they only responded at T1 with those responding at both T1 and T2, no significant differences were found concerning age, gender, years worked at the present unit or years worked in the profession.

**Table 2:** Participant characteristics

	*T1*	%	*T2*	%
Total number of participants, n	481		260	
Participants at public non-profit, n	407	85%	216	83%
Privately owned healthcare units, n	74	15%	44	17%
Women, n	422	88%	224	86%
Men, n	59	12%	35	13%
Profession, n				
-Registered Nurses	181	38%	101	39%
-Physician	70	15%	37	14%
-Administrator and secretary	64	13%	31	12%
-Physiotherapist	47	10%	30	12%
-Licensed Practical Nurse	45	9%	24	9%
-Social worker and psychologist	41	9%	21	8%
-Manager	26	5%	17	7%
-Occupational therapist	17	4%	8	3%
-Dietician	3	1%	1	< 1%
Age,				
Mean (SD)	50.2 (10.3)		50.6 (10.0)	
Md (Q_1−_Q_3_)	52.0 (44.0–59.0)		53.0 (44.0–59.0)	
Years worked at the present unit,				
Mean (SD)	9.1 (9.0)		9.3 (8.7)	
Md (Q_1−_Q_3_)	5.0 (2.0–14.0)		5.0 (3.0–14.0)	
Years worked in the profession,				
Mean (SD)	21.5 (12.1)		22.0 (11.8)	
Md (Q_1−_Q_3_)	21.0 (11.0–31.0)		20.0 (13.0–32.0)	

T1, data collection time 1; T2, data collection time 2; Md median; Q_1_-Q_3_, quartiles; SD standard deviation. Concerning participants in different professions, the numbers do not add up to 481 or 260 because some participants had multiple functions.

### Measures

All variables in the analyses – Lean maturity, care-giving, thriving, exhaustion, job demands and job resources – were measured using staff ratings. Ratings of Lean maturity reflect judgements regarding the entire unit. The ratings of care-giving, thriving, exhaustion and job demands and resources reflect judgements regarding their own situation. Staff perception of ***Lean maturity at their unit*** was measured using the 16-item Lean in Healthcare Questionnaire (LiHcQ; [48]. The questionnaire is based on Liker’s description of Lean and addresses Liker’s 4P: Philosophy, Processes, People and partners, and Problem-solving. Each item offers five response alternatives ranging from 1, indicating low maturity, to 5, indicating high maturity. In the study, a total score for the instrument was used, obtained by adding up the scores of all items in the questionnaire [48]. Thus, the possible range of total score is 16 to 80. The instrument has shown acceptable values for construct validity and internal consistency [48]. Cronbach’s alpha in the present study was 0.92.

Staff perception of their own ***job demands (JD)*** and ***job resources (JR)*** was assessed using Copenhagen Psychosocial Questionnaire II (COPSOQ II) [49], which is a valid, frequently used instrument [49–51]. Two overall indices were developed addressing job demands and job resources, inspired by the work of Bakker and Demeroutis [19, 20] (Table 3).

**Table 3:** Scales included in the job demands and job resources indices. Items were adopted from COPSOQ II [49]

	Scales included
Job Demands	Quantitative demands, (four items),
	Work pace (three items)
	Emotional demands (four items)
	Cognitive demands (four items)
	Role conflicts (four items)
Job Resources	Influence (four items)
	Possibilities for development (four items)
	Quality of leadership (four items)
	Social support from colleagues (three items)
	Social support from supervisors (three items)
	Predictability (two items)
	Recognition (three items)
	Role clarity (three items)
	Mutual trust between employees (three items)
	Variation (two items)

The items had different response alternatives, although most ranged from 1 (always; to a very large extent; all the time) to 5 (never/hardly never; to a very small extent; not at all). The scores 1–5 were recalculated to values from 0 to 100, where high scores indicate higher levels of job resources and demands. The average score was used for the indices [52]. In the present study, Cronbach’s alpha was 0.89 for both the job demands and job resources indices.

***Staff perception of satisfaction with their own provided care*** was measured using the Staff Satisfaction with Care (SSC) scale [53]. The SSC consists of nine items; we excluded one item concerning satisfaction with physical care, as we viewed primary care staff to be minimally involved in, e.g., helping patients with intimate hygiene or dressing. The items are rated on a 7-point scale, where 7 indicates the highest level of individual satisfaction regarding, e.g., the treatment, emotional engagement and support given to patients. The mean item score was used in further analyses. The SSC scale has been shown to be reliable [53]. Cronbach’s alpha in the present study was 0.91.

***Thriving*** was assessed on the basis of participants’ experience of their sense of learning and vitality, and measured using the 10-item thriving scale [41]. The scale includes five items each for the two factors. The response alternatives range from 1 (strongly disagree) to 7 (strongly agree), where 7 indicates the highest level of workplace thriving. The total scale was used and summarized as a mean score [41]. The scale has shown acceptable psychometric values [41]. Cronbach’s alpha in the present study was 0.90.

***Exhaustion*** was assessed using the 4-item burnout subscale in COPSOQ II [49]. The response alternatives range from 1 (all the time) to 5 (not at all), and the scores were recalculated to values from 0 to 100, with high scores indicating high levels of exhaustion. The mean score for the scale was used. The instrument has been validated with satisfying results [49, 50, 54] and use of only a few items to capture exhaustion has been shown to be reliable [55]. Cronbach’s alpha in the present study was 0.90.

### Statistical analysis

IBM SPSS Statistics, version 24, was used to describe the participants’ demographic data and to perform multivariate analysis on multilevel and repeated-measures data. For estimations of indirect effects, MLmed was used for moderated mediation and RMediation for mediation only.

#### Missing data

Missing data were dealt with using multiple imputation (MI), which makes use of all data regardless of the percentage of missing items [56]. MI replaces the missing data values and generates five imputed datasets together with the original dataset. A common recommendation [56, 57] is to use the same structure for MI as for the analyses; thus, three datasets were created based on each outcome variable (SSC, thriving and exhaustion) to test hypotheses H1 to H3 separately. Thereafter, MI was conducted for each of them. As a sensitivity analysis, we also analyzed data after deleting participants with more than 50% missing items.

#### ICC

Our design resulted in repeated data for individuals nested within primary care units. For guidance in selection of an analysis strategy, clustering effects within units were analyzed using intra-class correlation coefficient (ICC), with 95% CI. The clustering effects (ICC) ranged from 0.005 to 0.146 and decreased as all variables was added to the model (Table 4). Because clustering did not appear to be a major issue, the primary care unit level was excluded from the analysis of moderated mediation.

**Table 4:** ICC values for all three outcome variables and their associated mediators

	Only outcome, no covariates	With all the covariates^a^
*SSC*	0.023	0.023
Job resources	0.146	0.082
*Thriving*	0.005	0.007
Job resources	0.146	0.075
*Exhaustion*	0.034	0.026
Job demands	0.043	0.040

*ICC* intra-class correlation coefficient, *SSC* staff satisfaction with care

^a^The variables included in the model involving SSC were demands, gender and time. The variables included in the model involving thriving were demands, years worked in the profession and time. The variables included in the model involving exhaustion were resources, years worked in the profession and time

#### Test of H1, H2 and H3 – moderated mediation

The three hypotheses were tested using models involving moderated mediation. This comprised tests of whether changes over time in Lean maturity operated indirectly through a mediator (job resources or job demands) on an outcome (SSC, thriving and exhaustion), and whether the size of that indirect effect was dependent on (moderated by) another variable (job resources or job demands). The variables job demands and job resources were used as a mediator or moderator depending on the outcome variables (cf. Fig. 1), as described in the JD-R theory [19, 20]. Time was included as a covariate, as were gender and years worked in the profession. Regarding the covariates gender and years in the profession only statistically significant covariates were kept in the model. To model the nested and repeated structure of the data, the free SPSS macro program MLmed for multilevel mediation analysis [58] was used. As MLmed can only handle two levels (here the repeated design), the clustering of individuals within primary care units was ignored; this was also supported by the low ICC values (Table 4). MLmed was not developed for pooled datasets from MI, and therefore the last MI dataset was selected for analysis. An explicit quantification of moderated mediation was used: the index of moderated mediation [59]. Confidence intervals (CIs) were calculated using a Monte Carlo method [58].

#### Test of H1, H2 and H3 – mediation only

**Fig. 2:**
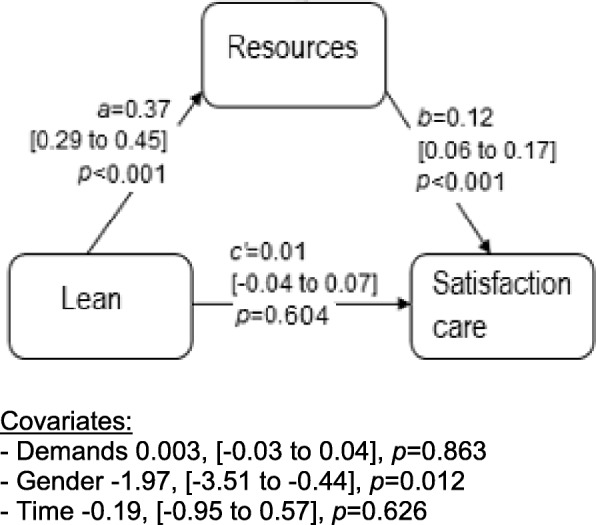
Direct (*c’*) and indirect effects (estimated by a*b) of the predictor Lean on the outcome variable staff satisfaction with care, mediated by job resources. Effects for covariates are shown below the figure. Within brackets, 95% confidence interval on the effect sizes

**Fig. 3:**
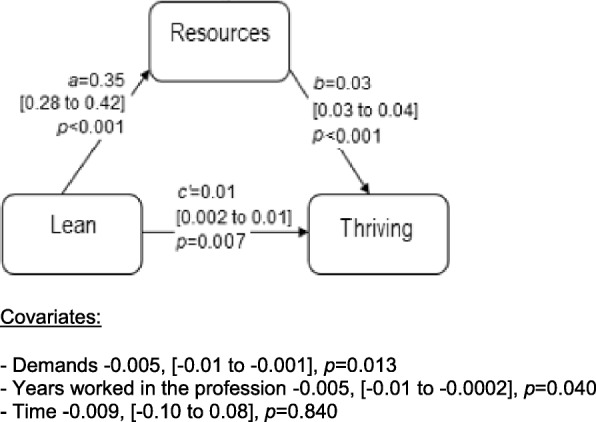
Direct (*c’*) and indirect effects (estimated by a*b) of the predictor Lean on the outcome variable thriving, mediated by job resources. Effects for covariates are shown below the figure. Within brackets, 95% confidence interval on the effect sizes

**Fig. 4:**
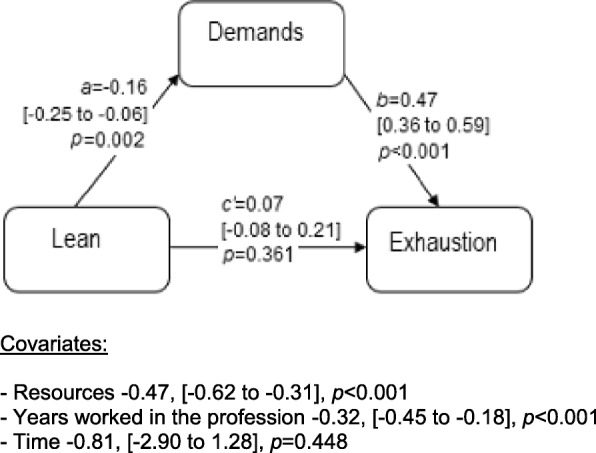
Direct (*c’*) and indirect effects (estimated by a*b) of the predictor Lean on the outcome variable exhaustion, mediated by job demands. Effects for covariates are shown below the figure. Within brackets, 95% confidence interval on the effect sizes

In a last step, guided by the tests involving moderated mediation, which showed that a moderating effect was not present, we proceeded to test our three hypotheses excluding moderators. RMediation [60], a stand-alone program based on R syntax, was used to test mediation. In the program, estimates of parameters and their standard errors are entered. For estimation of the direct effects and the components of indirect effects, we employed multilevel modelling using a linear mixed model [61]. In all three models, random intercepts with repeated measures were used to model clustered observations within units based on longitudinal data. A compound symmetry covariance structure was employed. Mediators in the models were job resources for SSC and thriving, and job demands for exhaustion. In addition to the control variables mentioned previously, we controlled for job demands or resources depending on the model tested. The indirect effect is estimated by the product of the effect of Lean maturity on the mediator and the effect of the mediator on the outcome, given the direct effect of Lean maturity and other covariates (Figs. 2, 3, and 4). Estimation of 95% CI of the indirect effects was made using the distribution of the product method in RMediation [60] .

Visual inspection of the residuals using histograms showed no major deviance from a normal distribution. A linear regression analysis, based on T1 data with all variables in the three models used for H1 to H3, indicated no multicollinearity; the highest variance inflation factor (VIF) value was 1.37. *P*-values < 0.05 were considered to indicate statistical significance, and 95% CIs were used to describe precision of estimates.

## Results

### Moderated mediation in the models associated with H1, H2 and H3

No support was found for job demands or job resources having a moderated mediation effect in the models addressing H1, H2, and H3 using the index of moderated mediation. The indices were small, and all 95% CIs included zero, SSC − 0.0023 to 0.0012, thriving − 0.0002 to 0.0002 and exhaustion − 0.0036 to 0.0016. Because no evidence of moderated indirect effects was found, we proceeded by testing only direct and indirect effects in all three models.

### Direct and indirect effects of Lean maturity on staff satisfaction with care (H1)

The results showed that, over time, changes in Lean maturity were associated with changes in staff satisfaction with care, indirectly through job resources (Fig. 2). This indirect effect was positive, meaning that when Lean maturity increased, staff satisfaction with care also increased. The indirect effect was estimated to 0.044 (95% CI 0.023 to 0.067). The direct effect (controlling for the mediator) was non-significant.

### Direct and indirect effects of Lean maturity on thriving (H2)

The results showed that, over time, changes in Lean maturity were associated with changes in thriving, indirectly through job resources (Fig. 3). This indirect effect was positive, meaning that when Lean maturity increased, thriving also increased. The indirect effect was estimated to 0.012 (95% CI 0.009 to 0.015). The direct effect (controlling for the mediator) was also significant.

### Direct and indirect effects of Lean on exhaustion (H3)

The results showed that, over time, changes in Lean maturity were associated with changes in exhaustion, indirectly through job demands (Fig. 4). This indirect effect was negative, meaning that when Lean maturity increased, exhaustion decreased. The indirect effect was estimated to − 0.07 (95% CI − 0.12 to − 0.03). The direct effect (controlling for the mediator) was non-significant.

### Sensitivity analysis

The sensitivity analysis showed only small differences in the results obtained regardless of whether all missing data were multiple imputed or whether we excluded participants with more than 50% missing items and imputated for the remaining participants (see Additional file 1).

## Discussion

The present study shows that when Lean maturity increased, staff satisfaction with care and thriving increased, mediated by increased job resources, whereas exhaustion decreased, mediated by decreased job demands. A relationship between increased Lean maturity and improved staff satisfaction with care has been seen earlier in our cross-sectional study in primary care [18], and similar results have been reported in other studies in the healthcare sector [12, 34]. Viewing Lean studies concucted at a hospital, an outpatient clinic and in primary care, the results show somewhat conflicting findings of satisfaction with care from the patient perspective [33, 35, 36]. This highlights the need to include different aspects of quality of care in future research. Concerning the results on Lean maturity and thriving, another study in primary care, reported similar findings, however addressing Lean and engagement, not thriving [11]. Our result on thriving was not unexpected, as Lean intends to increase resources, such as prerequisites for participation and learning. Having resources can contribute to staff engagement, motivation [19] and likely to thriving [23, 30]. To strive for increased thriving seems beneficial for both the individual staff and for the employer as thriving can contribute to improved job performance and staff health [35]. Concerning Lean maturity and exhaustion, our results indicate that Lean can be beneficial to staff health. Other studies, one conducted in primary care [11] and one included both hospitals and a municipality [24] have reported varied findings. A review of Lean in different sectors, including healthcare, supported our findings to some extent, as it showed that when Lean was adopted partially and used as a rationalization strategy, no improvements in health were observed [62]. However, if an organization is to achieve set goals, Liker suggested there must be systemwide adoption, including all of the Lean principles and implementation among all staff members [14]. This indicates the need for further research with a specific focus on Lean maturity and staff health in healthcare [1, 4, 9].

Job demands and job resources were not supported as moderated mediation variables in our models, although Bakker and Demerouti [19] described an interaction effect between them. However, including job demands and job resources as mediators, as suggested in the JD-R theory [19], played a significant role in all our models. Other factors previously found to influence outcomes, in studies focused mainly on auto and manufacturing industries as well as on healthcare, were how Lean was adopted [7, 63] and the organization’s traditions [7, 64]. These factors might, to some extent, explain the negative findings reported concerning Lean, working conditions and health [6–8]. Our positive findings might be influenced by, for instance, the traditions in Swedish healthcare, where staff are often involved in decision-making and problem-solving, which may facilitate Lean adoption and enhance positive findings [64]. One strength of our study was that we measured Lean maturity using LiHcQ, which measures all aspects of Lean [48]. Having staff as responders can be seen as both a strength and a limitation. It is a strength because staff have the best knowledge about, for instance, Lean adoption and their working conditions. It is a limitation because having staff as responders may introduce recall bias, their responses may reflect what is socially accepted, and they may be generally biased by a positive or negative attitude toward their workplace. Moreover, using the subjective measure of staff perception of satisfaction with care as an indication of quality of care may create bias, as it reflects only staff members view. Even more important is to include the patient perspective of quality of care as this is essential in Lean [14], and requires further attention [3, 65]. Testing moderated mediation and mediation was a strength, as this procedure broadens our understanding of a relationship between an independent and a dependent variable. Our longitudinal design is a strength; whereas the convenience sample and the low response rate [66] are limitations; both factors may influence the generalizability of our results. Our analyses were strengthened by dealing with missing data using multiple imputation followed by a sensitivity analysis, as recommended [56].

## Conclusions

The results indicate that primary care staff may benefit from working in organizations characterized by high levels of Lean maturity and that caregiving may also be improved as perceived by staff.

## Additional file

Additional file 1: Sensitivity analysis showing results from MI data. (DOCX 14 kb)

## Data Availability

The datasets generated and/or analysed during the current study are not publicly available due to the ethical approval who limits possibilities to share data. Data are only available to the researchers involved in this study.

## Abbreviations

4P: Philosophy, processes, people and partners, and problem-solving
CI: Confidence intervals
COPSOQ: Copenhagen Psychosocial Questionnaire
JD-R: Job demands- resources theory
LiHcQ: Lean in Healthcare Questionnaire
Md: Median
MI: Multiple imputation
SD: Standard deviation
SSC: Staff satisfaction with care
T1: Time 1
T2: Time 2

## References

[1] D’Andreamatteo A, Ianni L, Lega F, Sargiacomo M (2015). Lean in healthcare: a comprehensive review. Health Policy.

[2] De Souza LB (2009). Trends and approaches in lean healthcare. Leadersh Health Serv.

[3] Joosten T, Bongers I, Janssen R (2009). Application of lean thinking to health care: issues and observations. Int J Qual Health Care.

[4] Holden RJ (2011). Lean thinking in emergency departments: a critical review. Ann Emerg Med.

[5] Rees GH, Gauld R (2017). Can lean contribute to work intensification in healthcare?. J Health Organ Manag.

[6] Landsbergis PA, Cahill J, Schnall P (1999). The impact of lean production and related new systems of work organization on worker health. J Occup Health Psychol.

[7] Hasle P, Bojesen A, Jensen PL, Bramming P (2012). Lean and the working environment: a review of the literature. Int J Oper Prod Manag.

[8] Parker SK (2003). Longitudinal effects of lean production on employee outcomes and the mediating role of work characteristics. J Appl Psychol.

[9] Mazzocato P, Savage C, Brommels M, Aronsson H, Thor J (2010). Lean thinking in healthcare: a realist review of the literature. BMJ Qual Saf.

[10] Polit DF, Beck CT (2008). Nursing research: generating and assessing evidence for nursing practice.

[11] Hung DY, Harrison MI, Truong Q, Du X (2018). Experiences of primary care physicians and staff following lean workflow redesign. BMC Health Serv Res.

[12] Simons P, Backes H, Bergs J, Emans D, Johannesma M, Jacobs M (2017). The effects of a lean transition on process times, patients and employees. Int J Health Care Qual Assur.

[13] Pettersen J (2009). Defining lean production: some conceptual and practical issues. TQM J.

[14] Liker JK (2004). The Toyota way.

[15] Shah R, Ward PT (2007). Defining and developing measures of lean production. J Oper Manag.

[16] Spear S, Bowen HK (1999). Decoding the DNA of the Toyota production system. Harv Bus Rev.

[17] Womack JP, Jones DT, Roos D. The machine that changed the world: The story of Lean production. New York: Harper Collins; 1990.

[18] Kaltenbrunner M, Mathiassen SE, Bengtsson L, Engström M (2019). Lean maturity and quality in primary care. J Health Organ Manag.

[19] Bakker AB, Demerouti E (2017). Job demands–resources theory: taking stock and looking forward. J Occup Health Psychol.

[20] Bakker AB, Demerouti E (2007). The job demands-resources model: state of the art. J Manag Psychol.

[21] Bakker AB (2011). An evidence-based model of work engagement. Curr Dir Psychol Sci.

[22] Demerouti E, Bakker AB, Nachreiner F, Schaufeli WB (2001). The job demands-resources model of burnout. J Appl Psychol.

[23] Leiter MP, Bakker AB. Work engagement: a handbook of essential theory and research: New York: Psychology press; 2010.

[24] Lindskog P, Hemphälä J, Eklund J, Eriksson A (2016). Lean in healthcare: engagement in development, job satisfaction or exhaustion. J Hosp Admin.

[25] Gaither CA, Nadkarni A (2012). Interpersonal interactions, job demands and work-related outcomes in pharmacy. Int J Pharm Pract.

[26] Prem R, Ohly S, Kubicek B, Korunka C (2017). Thriving on challenge stressors? Exploring time pressure and learning demands as antecedents of thriving at work. J Organ Behav.

[27] Xanthopoulou D, Bakker AB, Demerouti E, Schaufeli WB (2007). The role of personal resources in the job demands-resources model. Int J Stress Manag.

[28] Clausen T, Nielsen K, Carneiro IG, Borg V (2012). Job demands, job resources and long-term sickness absence in the Danish eldercare services: a prospective analysis of register-based outcomes. J Adv Nurs.

[29] Gilboa S, Shirom A, Fried Y, Cooper C (2008). A meta-analysis of work demand stressors and job performance: examining main and moderating effects. Pers Psychol.

[30] Bakker AB, Van Veldhoven M, Xanthopoulou D (2010). Beyond the demand-control model. J Pers Psychol.

[31] Drotz E, Poksinska B (2014). Lean in healthcare from employees’ perspectives. J Health Organ Manag.

[32] Dellve L, Williamsson A, Strömgren M, Holden RJ, Eriksson A (2015). Lean implementation at different levels in Swedish hospitals: the importance for working conditions and stress. Int J Hum Factors Ergon.

[33] van L-ZJE, Brunings JW, Houkes I, van RAJA, Ruwaard D, Vrijhoef HJM (2016). Using lean thinking at an otorhinolaryngology outpatient clinic to improve quality of care. Laryngoscope..

[34] Yousri TA, Khan Z, Chakrabarti D, Fernandes R, Wahab K (2011). Lean thinking: can it improve the outcome of fracture neck of femur patients in a district general hospital?. Injury..

[35] Hwang P, Hwang D, Hong P (2014). Lean practices for quality results: a case illustration. Int J Health Care Qual Assur.

[36] Poksinska BB, Fialkowska-Filipek M, Engström J (2017). Does Lean healthcare improve patient satisfaction? A mixed-method investigation into primary care. BMJ Qual Saf.

[37] Van Bogaert P, Timmermans O, Weeks SM, van Heusden D, Wouters K, Franck E (2014). Nursing unit teams matter: impact of unit-level nurse practice environment, nurse work characteristics, and burnout on nurse reported job outcomes, and quality of care, and patient adverse events—a cross-sectional survey. Int J Nurs Stud.

[38] Westerberg K, Tafvelin S (2014). The importance of leadership style and psychosocial work environment to staff-assessed quality of care: implications for home help services. Heal Soc Care Community.

[39] Aiken LH, Clarke SP, Sloane DM, Consortium IHOR (2002). Hospital staffing, organization, and quality of care: cross-national findings. Int J Qual Health Care.

[40] Spreitzer G, Sutcliffe K, Dutton J, Sonenshein S, Grant AM (2005). A socially embedded model of thriving at work. Organ Sci.

[41] Porath C, Spreitzer G, Gibson C, Garnett FG (2012). Thriving at work: toward its measurement, construct validation, and theoretical refinement. J Organ Behav.

[42] Macey WH, Schneider B (2008). The meaning of employee engagement. Ind Organ Psychol.

[43] Radnor ZJ, Holweg M, Waring J (2012). Lean in healthcare: the unfilled promise?. Soc Sci Med.

[44] Maslach C, Jackson SE, Leiter MP, Schaufeli WB, Schwab RL (1986). Maslach burnout inventory.

[45] Aronsson G, Theorell T, Grape T, Hammarström A, Hogstedt C, Marteinsdottir I (2017). A systematic review including meta-analysis of work environment and burnout symptoms. BMC Public Health.

[46] Hakanen JJ, Bakker AB, Schaufeli WB (2006). Burnout and work engagement among teachers. J Sch Psychol.

[47] Anell A, Glenngård AH, Sweden MS (2012). Health system review. Health Syst Transit.

[48] Kaltenbrunner M, Bengtsson L, Mathiassen SE, Engström M (2017). A questionnaire measuring staff perceptions of Lean adoption in healthcare: development and psychometric testing. BMC Health Serv Res.

[49] Pejtersen JH, Kristensen TS, Borg V, Bjorner JB (2010). The second version of the Copenhagen Psychosocial Questionnaire. Scand J Public Health.

[50] Berthelsen H, Hakanen J, Kristensen T, Lönnblad A, Westerlund H. A qualitative study on the content validity of the social capital scales in the Copenhagen Psychosocial Questionnaire (COPSOQ II). Scand J Work Organ Psychol. 2016;1(1):1–13.

[51] Moncada S, Utzet M, Molinero E, Llorens C, Moreno N, Galtés A (2014). The Copenhagen psychosocial questionnaire II (COPSOQ II) in Spain—a tool for psychosocial risk assessment at the workplace. Am J Ind Med.

[52] Kristensen TS, Hannerz H, Høgh A, Borg V (2005). The Copenhagen Psychosocial Questionnaire-a tool for the assessment and improvement of the psychosocial work environment. Scand J Work Environ Health.

[53] Mårtensson G, Carlsson M, Lampic C (2010). Is nurse–patient agreement of importance to cancer nurses’ satisfaction with care?. J Adv Nurs.

[54] Berthelsen H, Lönnblad A, Hakanen J, Søndergård Kristensen T, Axtelius B, Bjørner JB (2014). Cognitive interviewing used in the development and validation of Copenhagen Psychosocial Questionnaire in Sweden.

[55] West CP, Dyrbye LN, Sloan JA, Shanafelt TD (2009). Single item measures of emotional exhaustion and depersonalization are useful for assessing burnout in medical professionals. J Gen Intern Med.

[56] Newman DA (2014). Missing data : five practical guidelines. Organ Res Methods.

[57] Black AC, Harel O, Betsy McCoach D (2011). Missing data techniques for multilevel data: implications of model misspecification. J Appl Stat.

[58] Rockwood NJ, Hayes AF (2017). MLmed: an SPSS macro for multilevel mediation and conditional process analysis. Poster presented at the annual meeting of the Association of Psychological Science (APS), Boston, MA.

[59] Hayes AF (2015). An index and test of linear moderated mediation. Multivar Behav Res.

[60] Tofighi D, MacKinnon DP (2011). RMediation: an R package for mediation analysis confidence intervals. Behav Res Methods.

[61] Fitzmaurice GM, Laird NM, Ware JH. Applied longitudinal analysis, vol. 998. Hoboken: Wiley; 2012.

[62] Westgaard RH, Winkel J (2011). Occupational musculoskeletal and mental health: significance of rationalization and opportunities to create sustainable production systems–a systematic review. Appl Ergon.

[63] Ulhassan W, von Thiele Schwarz U, Thor J, Westerlund H (2014). Interactions between lean management and the psychosocial work environment in a hospital setting – a multi-method study. BMC Health Serv Res.

[64] Sederblad P, Björkman T, Lundqvist K, Oudhuis M, Toivanen S, Landsbergis P (2013). Lean i arbetslivet.

[65] Moraros J, Lemstra M, Nwankwo C (2016). Lean interventions in healthcare: do they actually work ? A systematic literature review. Int J Qual Health Care.

[66] Baruch Y, Holtom BC (2008). Survey response rate levels and trends in organizational research. Hum Relat.

