# Supplementary material for: Staff perception of Lean, care-giving, thriving and exhaustion: a longitudinal study in primary care
Source: BMC Health Serv Res. 2019 Sep 9;19:652. doi: 10.1186/s12913-019-4502-6 (PMC6734292; doi:10.1186/s12913-019-4502-6)
Supplement: Supplementary file 1 — Sensitivity analysis showing results from MI data. (DOCX 14 kb) [file 12913_2019_4502_MOESM1_ESM.docx]

| **Additional file 1** Sensitivity analysis showing results from MI data | | | | |
| --- | --- | --- | --- | --- |
|  | Multiple imputation (pooled) | | Multiple imputation (>50%) (pooled) | |
| Parameter | Point estimate | 95% CI | Point estimate | 95% CI |
| *SSC* |  |  |  |  |
| Indirect effects | **0.044** | 0.023 to 0.067 | **0.043** | 0.021 to 0.067 |
| Direct effect (c´) | 0.014 | -0.038 to 0.065 | 0.021 | -0.029 to 0.071 |
| *Thriving* |  |  |  |  |
| Indirect effects | **0.012** | 0.009 to 0.015 | **0.013** | 0.010 to 0.016 |
| Direct effect (c´) | **0.008** | 0.002 to 0.013 | **0.008** | 0.002 to 0.013 |
| *Exhaustion* |  |  |  |  |
| Indirect effects | **-0.074** | -0.124 to -0.029 | **-0.067** | -0.117 to -0.022 |
| Direct effect (c´) | -0.067 | -0.076 to 0.210 | 0.118 | -0.035 to 0.272 |
| MI, multiple imputation; SSC, staff satisfaction with care; CI, confidence interval; >50%, those who responded to more than 50% of the items in the Lean in healthcare questionnaire | | | | |
